# Exploring risk for obstructive sleep apnea symptoms in vulnerable children: A cross-sectional analysis of clinical variables and oral health-related quality of life

**DOI:** 10.1590/1984-0462/2025/43/2025203

**Published:** 2025-12-12

**Authors:** Arnoldo Brasil Muniz, Patricia Rafaela dos Santos, Gabriell Bonifacio Borgato, Silvia Amélia Scudeler Vedovello, Diego Patrik Alves Carneiro

**Affiliations:** a Fundação Hermínio Ometto, Araras, SP, Brazil.; b Universidade Estadual de Campinas, Piracicaba, SP, Brazil.; c Centro Universitário Nossa Senhora do Patrocínio, Itu, SP, Brazil.

**Keywords:** Obstructive sleep apnea, Quality of life, Childrens, Social vulnerability, Apneia obstrutiva do sono, Qualidade de vida, crianças, Vulnerabilidade social

## Abstract

**Objective:**

The aim of the study was to verify the association between the presence of risk for obstructive sleep apnea (OSA), clinical variables, and oral health-related quality of life (OHRQoL) in children aged 3–10 years in a situation of social vulnerability.

**Methods:**

This observational, cross-sectional study included 400 children aged 3–10 years of both sexes enrolled in a municipal school in Itu, São Paulo. Children were in the deciduous or mixed dentition phase. Parents/guardians answered the Early Childhood Oral Health Impact Scale (ECOHIS) questionnaire and the Brazilian version of the Pediatric Sleep Questionnaire to assess OHRQoL and the risk of OSA. Clinical examinations were conducted by a calibrated examiner under natural lighting conditions. Dental caries were recorded using the decayed, missing, and filled teeth (deciduous teeth)/Decayed, Missing and Filled Teeth (permanent teeth) indexes, and malocclusion was recorded using the Foster and Hamilton criteria. Descriptive statistics and multiple logistic regression were performed to assess the association between the risk of OSA (dependent variable) and independent variables, including age, caries experience, malocclusion, and OHRQoL (ECOHIS), considering a significance level of 5%.

**Results:**

The prevalence of risk for OSA in the sample is 59.5%. Children over 7 years of age (odds ratio [OR] 6.02; 95% confidence interval (95%CI) 3.71–9.76) and with family OHRQoL impact (OR 1.68; 95%CI 1.07–2.65) are more likely to have a childhood of risk for OSA (p<0.05).

**Conclusions:**

Children in situations of social vulnerability over 7 years of age and with a negative impact on OHRQoL in the family domain have a greater chance of risk for OSA.

## RESUMO

**Objetivo:**

Verificar a associação entre a presença de risco para apneia obstrutiva do sono (AOS), variáveis clínicas e qualidade de vida relacionada a saúde bucal (QVRSB) em crianças de três a dez anos em situação de vulnerabilidade social.

**Métodos:**

Estudo observacional, transversal, com 400 crianças de três a dez anos de ambos os sexos, matriculadas em uma escola municipal de Itu, São Paulo. As crianças foram consideradas na fase de dentição decídua ou mista. Os pais/responsáveis responderam ao questionário Early Childhood Oral Health Impact Scale (ECOHIS) e à versão brasileira do Pediatric Sleep Questionnaire (PSQ) para avaliar a QVRSB e o risco de apneia obstrutiva do sono. Os exames clínicos foram realizados por examinador calibrado, em condições de iluminação natural. A cárie dentária foi registrada pelos índices ceo-d/CPOD, e a má oclusão, pelos critérios de Foster & Hamilton. Estatística descritiva e regressão logística múltipla foram realizadas para avaliar a associação entre o risco de apneia obstrutiva do sono (variável dependente) e variáveis independentes, incluindo idade, experiência de cárie, má oclusão e QVRSB (ECOHIS), considerando-se o nível de significância de 5%.

**Resultados:**

A prevalência de risco para AOS na amostra é de 59,5%. Crianças com mais de sete anos (*odds ratio* — OR 6,02; intervalo de confiança de 95% — IC95% 3,71–9,76) e com impacto na qualidade de vida com relação à saúde (OR 1,68; IC95% 1,07–2,65) apresentam maior probabilidade de ter uma infância de risco para AOS (p<0,05).

**Conclusões:**

Crianças em situação de vulnerabilidade social com mais de sete anos e com impacto negativo na qualidade de vida em relação à saúde no domínio familiar apresentam maior chance de risco para AOS.

## INTRODUCTION

Obstructive sleep apnea (OSA) is characterized by recurrent episodes of a partial or complete collapse of the upper airway during sleep, resulting in airflow interruption (apnea) or reduction (hypopnea), compromising sleep architecture and gas exchange.^1,2,3,4,5^This condition has been associated with cardiovascular and metabolic diseases, neurocognitive deficits, and behavioral problems.^2,3,6^ Its relationship with dental caries and periodontal disease has been reported, possibly linked to mouth breathing and related changes in salivary flow and composition that may increase oral dryness and cariogenic bacterial growth.^2,5,7,8^

Sleep-disordered breathing in children, which includes OSA and conditions such as habitual snoring, significantly affects not only the individual’s health and quality of life but also the family’s well-being and broader public health outcomes.^2,9^ These disorders are associated with neurocognitive impairments, behavioral problems, and learning difficulties in children.^10,11^ Early identification and intervention are thus crucial. Tamasas et al.^2^ demonstrated that oral health parameters in children diagnosed with OSA via polysomnography were comparable to those identified using the Pediatric Sleep Questionnaire (PSQ), with children at higher risk exhibiting a greater incidence of oral disease and poorer scores on oral health-related quality of life (OHRQoL) measures.

However, most studies on this topic have not focused on socially vulnerable populations, who are often at increased risk for both poor oral health and OSA, and may benefit most from early screening approaches.

This research was conducted in Itu, a medium-sized municipality in the state of São Paulo, Brazil, characterized by marked social inequalities. The selected school is located in a peripheral region with limited access to healthcare andeducational resources, making it representative of children living in conditions of high social vulnerability. Conducting the study in this context allows for the investigation of OSA symptoms in an underrepresented population that may be at increased risk and could benefit most from early screening approaches.

Given this gap, the present study aims to verify the association between the presence of risk for OSA, clinical variables, and OHRQoL in socially vulnerable children aged 3–10 years. We hypothesize that children at risk for OSA will present worse oral health indicators and more negative impacts on OHRQoL, especially in the family domain.

## METHOD

This school-based, cross-sectional study was designed and reported in accordance with the Strengthening the Reporting of Observational Studies in Epidemiology (STROBE) guidelines, and employed a census sample comprising children aged 3–10 years enrolled in a municipal public school located in a socially vulnerable area of Itu, São Paulo, Brazil. Data were collected between April/2024 and June/2024. A census approach was adopted to include all eligible students enrolled at the school, aiming to maximize statistical power, avoid sampling loss, and ensure representativeness within this specific vulnerable population.

Of the approximately 925 children enrolled at the school, 400 returned the signed informed consent form and completed all stages of the study. No missing data were observed among these participants, as all questionnaires were fully completed and all underwent clinical examination. Although the primary focus was on child-level outcomes, parents/guardians were considered part of the study population, as they provided essential data through standardized instruments. The school was selected because it is located in a socially vulnerable area of Itu and serves children from low-income families who rely almost exclusively on the public education and health systems, which makes it a representative setting for this population. Although only 400 (43%) children participated, comparisons between participants and non-participants indicated no differences in age distribution, suggesting that the analyzed sample was representative of the overall school population.

The municipality had an estimated population of 168,240 inhabitants in the year before the collection and a human development index (HDI) of 0.773 (Atlas of Human Development in Brazil).12 The EMTI Rede Saber IV school is located in a peripheral region of the municipality and serves approximately 925 children full-time.

Children of both sexes, with deciduous or mixed dentition, were included. Children with syndromes or conditions that caused physical and/or psychological limitations that prevented them from undergoing clinical examinations were excluded.

The study was approved by the Research Ethics Committee of the Nossa Senhora do Patrocínio University Center—CEUNSP (#73900823.7.0000.8287). The parents or legal guardians signed the Informed Consent Form (ICF) authorizing the children’s participation. The ICF was presented to and applied to the children, as provided for in national ethical guidelines.

The guardians answered the validated Brazilian version of the ECOHIS,^13^ consisting of 13 questions (nine related to the impact on the child and four to the impact on the family). The answers ranged from 0 to 5 (0=never; 1=rarely; 2=occasionally; 3=often; 4=very often; 5=do not know). The total score ranged from 0 to 52. Following previous studies,^14,15^ scores were dichotomized according to the sample median, with values below the median classified as lower impact on quality of life and values above the median as higher negative impact.

The risk of OSA was assessed using the Brazilian version of the PSQ.^16,17^ The instrument consists of 22 questions divided into three domains (snoring, sleepiness, and behavior). Affirmative answers received a score of 1; negative answers and “I don’t know” received 0. Consistent with the original validation study,^16,17^ a cutoff point of ≥8 affirmative answers was adopted to indicate suspected OSA in children.

Clinical examinations were performed on the school premises, in a room with natural lighting, wooden spatulas, and gauze, by a single previously trained and calibrated examiner. The participation process involved a theoretical stage and a practical stage, with a 7-day interval between them. An intra-examiner agreement presented Kappa values of 0.89 for dental caries and 0.92 for malocclusion. Inter-examiner agreement was 0.87 for caries and 0.90 for malocclusion.

The presence of caries was assessed according to the criteria and codes of the World Health Organization^18^ using the dmft/DMFT indexes. Caries experience was dichotomized: absent (dmft/DMFT=0) or present (dmft/DMFT>0).^15,19,20^

Malocclusion was examined according to the criteria of Foster and Hamilton.^21^ Overjet was considered the relationship of the incisors in the horizontal direction. No distance between the maxillary and mandibular incisors was defined as normal overjet (0 mm). Increased overjet was recorded when the distance was >2 mm, and previous crossbite was recorded when the distance was <0 mm. Normal overbite was defined when the maxillary incisors overlapped the mandibular incisors by 2 mm. An overbite greater than 2 mm was designated as a deepoverbite. Anterior crossbite was recorded when the mandibular incisors were observed in front of the maxillary incisors. The anterior open bite was recorded when there was no contact between the anterior teeth when the posterior teeth were in occlusion. Posterior crossbite was recorded when the maxillary primary molars were occluded in lingual relation to the mandibular primary molars in centric occlusion. School children were diagnosed with the absence of malocclusion when all conditions were normal. When exhibiting at least one of the conditions mentioned above, they were classified as having an absence of malocclusion.^20,22^

Data analyses were performed using the R program, adopting a significance level of 5%. Descriptive analyses of the data were performed, presenting absolute and relative frequencies. Subsequently, logistic regression analyses were conducted to assess the association between each independent variable and the outcome of sleep apnea, estimating the raw odds ratios with their respective confidence intervals (95% confidence interval [95%CI]). The variables that presented p<0.20 in the individual analyses were selected to be studied in the multiple logistic regression model. In the final model, the variables with p≤0.05 remained after adjustments for the other variables, and the adjusted odds ratios with 95%CI were estimated. The model fit was assessed by the statistical significance of the estimates, the Akaike information criterion (AIC), -2 Log Likelihood, and the Hosmer and Lemeshow test.

## RESULT

Table 1 presents the results of the data analysis. The prevalence of childhood sleep apnea in the sample is 59.5%. When analyzed individually, age, caries experience, child, family, and total ECOHIS scores showed a significant association with sleep apnea (p<0.05). Analyzing these variables together in the multiple model, only age and family ECOHIS scores remained significant (p<0.05). Although child, family, and total ECOHIS scores were individually associated with OSA in the bivariate analysis, only ECOHIS (child) and ECOHIS (family) were tested simultaneously in the multiple model. ECOHIS (total) was not included because it represents the sum of the other two domains and could introduce collinearity.

**Table 1.** Analyses (crude and adjusted) of associations between the PSQ (suggestive of childhood sleep apnea) and demographic, clinical, and oral health impact variables (n=400).

Variable	Category	n (%)	Risk of obstructive sleep apnea (PSQ)		OR crude (95%CI)	p-value	OR adjusted (95%CI)	p-value
			Absent	Presence*				
			n (%)	n (%)				
Global sample	—	400 (100.0)	162 (40.5)	238 (59.5)	—	—	—	—
Sex	Female	209 (52.2)	84 (40.2)	125 (59.8)	1.03 (0.69-1.53)	0.8953	_—_	_—_
	Male	191 (47.8)	78 (40.8)	113 (59.2)	1			
Age	<7	236 (59.0)	133 (56.4)	103 (43.6)	1		1	
	>7	164 (41.0)	29 (17.7)	135 (82.3)	6.01 (3.73-9.68)	<0.0001	6.02 (3.71-9.76)	<0.0001
Caries experience	Absent	207 (51.7)	95 (45.9)	112 (54.1)	1		—	—
	Presence	193 (48.3)	67 (34.7)	126 (65.3)	1.58 (1.05-2.36)	0.0267		
Malocclusion	Absent	61 (15.3)	25 (41.0)	36 (59.0)	1			
	Presence	339 (84.8)	137 (40.4)	202 (59.6)	1.02 (0.60-1.78)	0.9333	—	—
ECOHIS (Child)	<1 (median)	219 (54.8)	102 (46.6)	117 (53.4)	1		_—_	_—_
	>1	181 (45.3)	60 (33.1)	121 (66.9)	1.76 (1.17-2.64)	0.0067		
ECOHIS (Family)	<0 (median)	240 (60.0)	111 (46.3)	129 (53.8)	1		1	
	>0	160 (40.0)	51 (31.9)	109 (68.1)	1.84 (1.21-2.79)	0.0043	1.68 (1.07-2.65)	0.0248
ECOHIS (Total)	<2 (median)	230 (57.5)	106 (46.1)	124 (53.9)	1		_—_	_—_
	>2	170 (42.5)	56 (32.9)	114 (67.1)	1.74 (1.15-2.63)	0.0084		

* Outcome event. 1: Reference category for independent variables.

OR: odds ratio; CI: confidence interval; ECOHIS: Early Childhood Oral Health Impact Scale — impact of oral health on quality of life (higher values indicate greater impact); AIC (empty model): 541.99; AIC (final model): 477.76; -2 Log L (empty model): 539.99; -2 Log L (final model): 471.76; Hosmer and Lemeshow test p: 0.8212.

Older children, aged over 7 years (odds ratio [OR] 6.02; 95% CI 3.71–9.76) and with an impact of oral health on the family’s quality of life (OR 1.68; 95%CI 1.07–2.65), are more likely to have childhood sleep apnea (p<0.05). It is observed that the prevalence of sleep apnea is 43.6% among children up to 7 years old and 82.3% among children over 7 years old. In addition, the prevalence is 53.8% among children without an impact of oral health on the family’s quality of life and 68.1% among children with some impact (Table 1).

## DISCUSSION

OSA is a common condition in children that significantly affects their general health and specific quality of life (OHRQoL). This study sought to investigate whether clinical oral health conditions and OHRQoL are associated with symptoms suggestive of OSA in children in socially vulnerable contexts. Our results indicated a statistically significant association between the presence of OSA symptoms and a negative impact on OHRQoL in the family domain.

The literature documents a wide variation in OSA prevalence rates, ranging from 9.5 to 51.9% when assessed by the PSQ.^23,26^ Our study observed a prevalence of 59.5%, indicating a substantial proportion of the sample with suspected OSA.

Regarding OHRQoL, children with suspected OSA presented significantly higher scores in the family domain. This finding suggests an association between the presence of OSA symptoms and a greater perceived burden on family life. The literature supports this observation, indicating that factors such as sleep disturbances caused by snoring, financial costs related to treatment, and the emotional stress experienced by parents or guardians may contribute to a more negative perception of family well-being.^27,28^

The study also found a significant association between OSA symptoms and age, being more prevalent in children aged seven years or older. The literature indicates that while younger children may experience more severe forms of OSA due to anatomical and physiological characteristics, older children continue to be affected by risk factors such as obesity and adenotonsillar hypertrophy, which maintain high prevalence levels.^29,30,31^

Although the association between OSA and oral health variables such as malocclusion and caries was not statistically significant in the final model, the literature suggests a strong biological plausibility. Mouth breathing, a common symptom in OSA, is associated with alterations in craniofacial development and salivary function, contributing to both malocclusion and increased caries risk.^2^ In our sample, 59.6% of children with malocclusion and 65.3% with caries experience had scores suggestive of OSA, reinforcing the need for further longitudinal investigation on these associations.

As strengths, the study used validated tools (PSQ and ECOHIS) and focused on a socially vulnerable population, a group underrepresented in OSA-related research. However, social vulnerability was inferred from the geographic location of the school, without direct measurement of individual socioeconomic indicators such as household income or parental education, which may have introduced uncontrolled heterogeneity within the sample. Another limitation concerns the use of the PSQ as a screening instrument rather than a diagnostic tool. Although validated and widely employed, it does not replace clinical evaluation or polysomnography, and this should be considered when interpreting the findings.

From a clinical and public health perspective, our findings underscore the importance of incorporating sleep-related questions into pediatric dental anamnesis, particularly in primary care settings. Low-cost screening instruments such as the PSQ can be integrated into school-based health initiatives and the Brazilian Programa Saúde na Escola, facilitating early detection of children at risk for OSA. Interdisciplinary approaches involving pediatric dentistry, speech-language pathology, pediatrics, and family health teams may enhance timely referral and comprehensive management, ultimately improving outcomes for children in socially vulnerable contexts

In conclusion, children in situations of social vulnerability over 7 years of age and with a negative impact on OHRQoL in the family domain were more likely to present symptoms suggestive of OSA.

## Data Availability

The database that originated the article is available with the corresponding author.
